# Beyond Absolute Blood Pressure Targets During Endovascular Thrombectomy: A Narrative Review of Intraprocedural Hemodynamics

**DOI:** 10.7759/cureus.117604

**Published:** 2026-10-07

**Authors:** Rajesh Veeramachaneni, Gadidesi Kalyani, Revathi Lokala, Abhishek Prasad, Nishank Patel Nooli, Raja Shekar Jadav, Sai Abhinay Reddy, Vincent Burton

**Affiliations:** 1 Anesthesiology and Critical Care, Rand Memorial Hospital, Freeport, BHS; 2 Anesthesiology and Critical Care, Sri Venkateswara Institute of Medical Sciences, Tirupati, IND; 3 Anesthesiology, Rand Memorial Hospital, Freeport, BHS; 4 Anesthesiology, University of Texas (UT) Health Medical Center, Houston, USA; 5 Anesthesiology and Perioperative Medicine, University of Alabama at Birmingham Heersink School of Medicine, Birmingham, USA; 6 Internal Medicine, Cheyenne Regional Medical Center, Cheyenne, USA; 7 Internal Medicine, Rajiv Gandhi Institute of Medical Sciences, Adilabad, IND

**Keywords:** acute ischemic stroke, anesthetic management, blood pressure, cerebral autoregulation, conscious sedation, endovascular thrombectomy, general anesthesia, reperfusion

## Abstract

Hemodynamic management is a central component of procedural care for acute ischemic stroke (AIS) during endovascular thrombectomy (EVT). Current guideline recommendations predominantly provide absolute blood pressure (BP) targets for intraprocedural management. Studies have also examined relative BP reduction, the magnitude and duration of hypotensive exposure, and blood pressure variability (BPV), providing additional measures of systemic hemodynamic exposure during EVT. However, definitions, baseline reference pressures, measurement intervals, and BPV metrics vary widely across studies, and associations with neurological outcomes are inconsistent. Importantly, these systemic BP parameters do not directly establish cerebral perfusion. This limitation supports investigating cerebral autoregulation as a complementary, patient-specific assessment. Evidence linking individualized autoregulatory limits to outcomes remains predominantly post-EVT, with very limited evidence during the pre-recanalization phase under anesthesia.

This review examines intraprocedural BP during EVT under anesthesia beyond absolute pressure targets. It considers relative BP reduction, the magnitude and duration of hypotensive exposure, pressure-time burden, BPV, and individualized autoregulatory limits in relation to cerebral perfusion. A narrative literature search was conducted in PubMed for studies evaluating hemodynamics during EVT under anesthesia, supplemented by screening of selected reference lists. Studies examining intraprocedural BP targets, relative changes, hypotension duration, pressure-time burden, and BPV in relation to neurological or imaging outcomes under anesthesia were prioritized, with emphasis on the pre-recanalization period. Post-EVT hemodynamic studies were not included in the primary synthesis, except where they informed the discussion of cerebral autoregulation and future research.

Intraprocedural hemodynamic variables during EVT characterize different aspects of systemic arterial-pressure exposure that may be relevant to cerebral perfusion in the ischemic penumbra. Absolute pressure, relative change, magnitude and duration of hypotension, pressure-time burden, and BPV should be regarded as complementary rather than interchangeable measures. Differences in reference pressures, definitions, measurement intervals, exposure periods relative to recanalization, and anesthesia techniques limit comparisons and standardization across studies. Future prospective studies, particularly during general anesthesia (GA), should integrate clearly defined systemic BP metrics with dynamic cerebral autoregulation. This may help determine which parameters or combinations best reflect cerebral physiological vulnerability. Individualized autoregulation-guided BP targets remain investigational and require prospective validation before they can inform routine clinical hemodynamic management.

## Introduction and background

Endovascular thrombectomy (EVT) has transformed the treatment of acute ischemic stroke (AIS) caused by large-vessel occlusion [1]. Until recanalization is achieved, however, the ischemic penumbra remains dependent on residual and collateral cerebral blood flow. In this setting, systemic blood pressure (BP) is a potentially modifiable determinant of cerebral perfusion, particularly when autoregulatory capacity is impaired [2,3]. Anesthetic management may further influence this relationship through induction-related hypotension, vasodilation, alterations in carbon dioxide tension, and the use of vasoactive agents. Consequently, maintaining adequate hemodynamics during EVT is an important component of anesthetic management [2].

The 2026 American Heart Association / American Stroke Association (AHA/ASA) guideline recommends maintaining BP ≤180/105 mmHg during EVT, although the optimal intraprocedural BP range, particularly the lower limit before recanalization, remains uncertain [4]. Randomized anesthesia strategy trials like SIESTA (Sedation vs Intubation for Endovascular Stroke Treatment), ANSTROKE (Anesthesia During Stroke), and GOLIATH (General or Local Anesthesia in Intra Arterial Therapy) incorporated protocolized intraprocedural SBP targets, generally ranging from 140 to 180 mmHg [5-7]. These trials contributed important prospective evidence on hemodynamic management during EVT within different anesthetic strategies.

This uncertainty regarding the lower limit of intraprocedural BP is particularly relevant in the pressure-dependent ischemic circulation. Favorable collateral circulation is associated with preservation of ischemic territory, but collateral-dependent perfusion may remain vulnerable to reductions in systemic pressure when autoregulatory reserve is impaired in stroke patients [2]. Thus, systolic blood pressure (SBP) or mean arterial pressure (MAP) that remains within an accepted absolute range may nevertheless represent a substantial reduction from an individual patient's baseline pressure. Conversely, an isolated BP nadir may not adequately represent a prolonged but less severe reduction in pressure. These considerations have led investigators to characterize intraprocedural hemodynamics using measures beyond absolute BP, including the magnitude of reduction from baseline, percentage change, cumulative and continuous duration below pressure thresholds, pressure-time burden, and blood pressure variability (BPV) [8-10].

Observational evidence supports the relevance of these additional dimensions, although findings have not been uniform. Relative reductions in MAP have been associated with unfavorable functional outcomes across several EVT cohorts, while measures that integrate the magnitude and duration of hypotension have further characterized hemodynamic exposure. Petersen et al., for example, demonstrated that mean procedural MAP could be similar between outcome groups despite significant differences in relative BP reduction and hypotensive burden [9]. Other studies have examined the frequency and duration of hypotensive episodes, area under threshold (AUT) measures, and BPV [8,11,12]. At the same time, differences in the definition of baseline BP, hemodynamic metrics, measurement intervals, anesthesia technique, and timing relative to recanalization complicate interpretation across studies. A systematic review of early EVT literature similarly identified substantial heterogeneity in BP monitoring and definitions, which precluded quantitative synthesis [8].

Dynamic cerebral autoregulation (dCA) provides a complementary physiological framework for interpreting these observations. When autoregulatory capacity is preserved, cerebral blood flow can be maintained across changes in systemic pressure. This buffering capacity may be impaired after AIS, with heterogeneous autoregulatory limits between patients. Consequently, the lower limit of autoregulation (LLA) may represent a patient-specific pressure boundary below which cerebral perfusion becomes increasingly pressure dependent [13]. Emerging studies relating BP excursions below individualized autoregulatory limits to infarct progression therefore raise the possibility that future intraprocedural targets could incorporate patient-specific cerebrovascular physiology alongside conventional systemic BP measures [14]. Such approaches remain investigational and require prospective validation. The updated European Stroke Organization (ESO) guideline highlights real-time cerebral hemodynamic monitoring and autoregulation-guided thresholds as potential directions for future individualized BP management [15].

This narrative review examines intraprocedural BP management during EVT for AIS, with particular emphasis on the period before recanalization. Evidence from EVT performed under general anesthesia (GA), conscious sedation (CS), and monitored anesthesia care is considered broadly, with the present review focusing on intraprocedural BP characteristics without separately evaluating the hemodynamic effects of specific anesthetic agents or individual vasopressors. The primary objective is to determine whether absolute BP targets alone adequately characterize intraprocedural hemodynamic exposure and to evaluate how relative BP reduction, magnitude and duration of hypotension, pressure-time burden, BPV, and individualized autoregulatory assessment may provide complementary information relevant to cerebral perfusion and neurological outcome. Cerebral autoregulation is considered an investigational physiological framework and future research direction rather than a currently validated intraprocedural therapeutic target.

## Review

Literature search and review methodology

A narrative literature review was conducted to identify studies evaluating intraprocedural blood pressure and hemodynamic characteristics during endovascular thrombectomy for acute ischemic stroke. PubMed was searched for English-language literature on human studies published predominantly from 2015 onward, representing the contemporary EVT era. Earlier studies were included when directly relevant to intraprocedural hemodynamics or cerebral autoregulation. The last literature search was conducted on June 28th, 2026. Search terms included combinations of “acute ischemic stroke,” “endovascular thrombectomy,” “mechanical thrombectomy,” “blood pressure,” “hypotension,” “mean arterial pressure,” “blood pressure reduction,” “blood pressure variability,” “hypotensive burden,” “cerebral autoregulation,” “collateral circulation,” “general anesthesia,” and “conscious sedation.” Reference lists of relevant studies and reviews were also screened for additional publications.

Randomized trials, secondary or post hoc analyses, and observational studies that reported BP or hemodynamic exposure during EVT were considered. Conference abstracts were considered when they provided clinically relevant recent data that had not yet appeared as a full peer-reviewed publication and were explicitly identified as preliminary evidence in the text. Their inclusion was intended to capture emerging evidence relevant to the scope of this narrative review. Studies were selected when they provided data on absolute BP targets, relative BP reductions, magnitude and duration of hypotension, pressure-time burden, BPV, and individualized autoregulatory measures. As definitions of baseline BP and hemodynamic parameters differed across studies, each was reported according to the definition used by the original investigators. Reference BP is used as the overarching term for the different reference pressures used across studies. Titles and abstracts were reviewed for relevance to the predefined hemodynamic domains, followed by full-text assessment of potentially relevant studies. Study inclusion was purposive for this narrative synthesis and did not follow a formal screening protocol.

Evidence from EVT performed under GA, CS, and monitored anesthesia care was considered. The review focused on intraprocedural BP characteristics and did not separately evaluate the effects of specific anesthetic agents or individual vasopressors. Studies evaluating BP after recanalization or following EVT were excluded, except when relevant to the evaluation of cerebral autoregulation. Studies were grouped according to the hemodynamic parameter evaluated: absolute BP targets, magnitude and duration of BP reduction, BPV, and individualized cerebral autoregulation.

Physiological basis for pressure-sensitive cerebral perfusion during EVT

Cerebral ischemia following large-vessel occlusion represents a dynamic continuum of tissue injury, ranging from less severely hypoperfused oligemia and potentially salvageable penumbra to irreversibly injured ischemic core [16]. Before recanalization, preservation of penumbral tissue depends on residual antegrade and collateral blood flow. Collateral circulation can slow infarct progression, but its effectiveness is influenced by the pressure gradient supporting flow and by the remaining cerebrovascular autoregulatory reserve.

Dynamic cerebral autoregulation (dCA) describes the cerebrovascular response to spontaneous or induced changes in arterial pressure over time. It can be assessed by relating arterial-pressure fluctuations to simultaneous changes in cerebral blood-flow velocity using transcranial Doppler (TCD) or to cerebral oxygenation signals using near-infrared spectroscopy (NIRS) [17]. In acute ischemic stroke, autoregulatory buffering may be impaired and heterogeneous across patients. When autoregulatory reserve is reduced, systemic pressure reductions or fluctuations may therefore have greater effects on collateral-dependent cerebral perfusion. This provides a physiological rationale for considering not only guideline-recommended BP targets during EVT but also the magnitude, duration, and variability of intraprocedural pressure changes. The BPV, for example, has been associated with 90-day functional outcomes in several studies [12] and represents a dimension of hemodynamic exposure not captured by absolute BP or hypotensive burden alone.

Studies have commonly employed SBP targets of 140 to 180 mmHg during EVT [8]. However, the magnitude of pressure reduction that can be tolerated relative to an individual patient’s baseline remains uncertain, particularly in the setting of impaired cerebral autoregulation. Beyond absolute and relative pressure reductions, emerging evidence indicates that the temporal and dynamic characteristics of systemic BP may also be associated with neurological outcome [9,11]. For interpretation, intraprocedural systemic BP was considered across four related but non-equivalent domains: (i) absolute pressure, (ii) relative change from a defined reference pressure, (iii) magnitude and temporal burden of hypotension, and (iv) BPV.

Intraoperative hemodynamic metrics

Absolute BP

Earlier randomized anesthesia-strategy trials commonly incorporated protocolized intraprocedural SBP ranges between approximately 140 and 180 mmHg. The SIESTA, ANSTROKE, and GOLIATH trials showed that protocolized GA could be performed for EVT, with outcomes comparable to CS [5-7]. These trials used lower SBP protocol boundaries near 140 mmHg (SIESTA: 140-160 mmHg; ANSTROKE: 140-180 mmHg; GOLIATH: ≥140 mmHg), indicating that an SBP near 140 mmHg has been commonly incorporated as a lower protocol boundary in randomized anesthesia-strategy trials. These studies were not designed to establish a lower physiological limit for cerebral perfusion. Rasmussen et al. subsequently pooled 365 patients from the SIESTA, ANSTROKE, and GOLIATH trials and recharacterized BP exposure by incorporating relative reductions and duration below absolute MAP thresholds. Increasing delta MAP (dMAP) and cumulative exposure to MAP <70 mmHg for ≥10 minutes were associated with worse 90-day functional outcome [18]. Thus, maintenance within an absolute SBP range and preservation of pressure relative to a reference baseline represent distinct dimensions of systemic hemodynamic exposure.

In the original GOLIATH trial, GA was associated with lower SBP and MAP, more frequent >20% reductions in MAP, and greater vasopressor use than CS. However, these hemodynamic differences were not associated with worse neurological outcomes with GA. The favorable outcome signal with GA was considered by the authors to be partly related to higher reperfusion success [7]. In a subsequent secondary analysis of GOLIATH, Rasmussen et al. examined these hemodynamic differences and found no significant association between the evaluated BP parameters and 90-day functional outcomes. Notably, despite more frequent hypotensive episodes with GA, MAP<80 mmHg, when present, was longer with CS [19]. These observations add to the evolving evidence that the occurrence, magnitude, and duration of BP reductions represent distinct dimensions of intraprocedural hemodynamic exposure.

In a systematic review of nine studies involving 1,037 patients, Maïer et al. found considerable heterogeneity in intraprocedural BP assessment, precluding quantitative synthesis. MAP was most frequently used to characterize intraprocedural BP changes, whereas SBP was predominantly used to define periprocedural targets. Studies using strict SBP targets of 140-180 mmHg did not demonstrate an association between SBP and functional outcomes, whereas other studies identified different patterns of BP reduction as predictors of unfavorable outcomes [8]. These findings demonstrate that intraprocedural hemodynamic exposure has been characterized using different parameters and support evaluating these measures individually to determine their respective relationships with cerebral perfusion and neurological outcome during EVT.

The SBP and MAP should therefore be interpreted as related but distinct hemodynamic measures. The SBP is convenient for protocolized targets, whereas MAP represents mean systemic arterial driving pressure and is frequently used to quantify intraprocedural reductions. Neither measure alone directly establishes cerebral perfusion pressure, which is also influenced by intracranial pressure, vascular resistance, collateral anatomy, and autoregulatory state.

At the time of this narrative review, the MASTERSTROKE (management of systolic blood pressure during thrombectomy by endovascular route for acute ischemic stroke) trial had reported results as a late-breaking conference abstract, with full publication awaited. The full peer-reviewed report was not available at the time of this review. The trial randomized 562 patients undergoing EVT under GA to intraprocedural SBP targets of 140 ± 10 or 170 ± 10 mmHg until recanalization. Preliminary results showed no significant difference in 90-day functional outcomes, providing no evidence of additional benefit from augmenting SBP to the higher target within the range studied [20]. These preliminary findings should not be interpreted as establishing 140 ± 10 mmHg as an optimal target or as a validated lower safety boundary; the lower intraprocedural pressure limit before recanalization remains unresolved.

Table 1 summarizes selected studies evaluating intraprocedural blood pressure within protocolized or randomized absolute BP targets during EVT. Collectively, these findings suggest that absolute SBP targets may be complemented by assessing the magnitude and duration of pressure deviations, providing a broader characterization of hemodynamic exposure during EVT under anesthesia. Importantly, several of these analyses provide additional prognostic information from the same randomized cohorts. Such secondary analyses should be interpreted as hypothesis-generating and should not be considered independent randomized evidence.

**Table 1:** Studies evaluating intraprocedural BP within protocolized or randomized absolute BP targets during EVT Note: Studies are categorized according to the primary BP-management framework. Additional hemodynamic parameters are reported where applicable. Study-specific definitions: dMAP = baseline MAP − mean procedural MAP; ARV = mean absolute difference between consecutive BP measurements. ARV: Average real variability, BP: Blood pressure, BPV: Blood pressure variability, CS: Conscious sedation, DBP: Diastolic blood pressure, dMAP: Delta mean arterial pressure, EVT: Endovascular thrombectomy, GA: General anesthesia, MAP: Mean arterial pressure, MABP: Mean arterial blood pressure, mRS: Modified Rankin scale, NIHSS: National Institutes of Health Stroke Scale, NS: Not specified, SAGA: SIESTA, ANSTROKE, and GOLIATH association, SBP: Systolic blood pressure

Study	Absolute BP protocol/target	Baseline/ reference BP	Hemodynamic parameter	Study findings and hemodynamic interpretation
Schönenberger et al., 2018 (post hoc SIESTA analysis) [21]	Randomized GA vs CS, protocolized BP management 140-160 mmHg target	Admission BP (single brachial measurement)	Changes in SBP, DBP and MAP from baseline across procedural phases	BP reductions were not associated with 24-hour N ational Institutes of Health Stroke Scale ( NIHSS) or three-month modified Rankin Scale (m RS). Baseline DBP and MAP in CS showed associations with early NIHSS change.
Löwhagen Hendén et al., 2017 (ANSTROKE) [6]	Randomized anesthetic strategy, BP protocolized; SBP 140-180 mmHg before recanalization in both GA and CS groups	Last recorded MAP before induction of anesthesia	SBP, DBP, MAP, >20% and >40% MAP reduction from baseline, duration below thresholds	No difference in BP decline between GA and CS, no difference in 90-day mRS.
Simonsen et al., 2018 (GOLIATH) [7]	Randomized anesthetic strategy, BP protocolized. SBP>140 mmHg, MAP>70 mmHg	Preinduction BP	SBP/MAP, >20% MAP decline, MAP <70 mmHg and duration	GA had lower SBP/MAP and more >20% MAP reductions, without worse functional outcome.
Campbell et al., 2026 (MASTERSTROKE) [20]	Randomized SBP 140±10 vs 170±10 mmHg during GA until recanalization	Not specified (NS)	NS	No significant difference in 90-day mRS between SBP targets.
Rasmussen et al. (pooled analysis of SIESTA, ANSTROKE and GOLIATH (SAGA)), 2020 [18]	SBP<140 mmHg, mean arterial blood pressure (MABP)<70, <80 and <90 mmHg, minimum SBP/MABP	Last BP before induction.	Baseline/procedural SBP & MABP, >20% BP reduction, dMAP, minimum/maximum BP, MABP thresholds, cumulative and continuous threshold duration	MABP<70 mmHg for ≥10 cumulative min or ≥20 continuous min, and MABP>90 mmHg for ≥45 cumulative min or ≥115 continuous min was associated with worse 90-day mRS. Prognostic information associated with an absolute threshold may depend on duration of exposure.
Rasmussen et al., 2015 (GOLIATH secondary analysis) [19]	GA/CS. SBP>140, MAP>70 mmHg	Preinduction BP	>20% MAP reduction, time MAP <90/<80/<70, time SBP <140, BPV (dMAP, ARV_syst,diast, MAP_)	Multiple BP dimensions differed significantly between GA and CS, but none independently predicted 90-day mRS.
Löwhagen Hendén et al., 2015 [22]	NS	Preinduction MAP	>20%/>40% MAP decline from baseline, occurrence and duration	>40% MAP decline independently associated with poor outcome. Duration was not significant.
Deng et al., 2020 [23]	Randomized SBP 130-150 vs 160-180 mmHg, from start of anesthesia to recanalization	NS	Achieved SBP, feasibility of target separation, clinical outcomes	Targets produced distinct mean SBP (139 vs 167 mmHg). No significant difference in 90-day functional independence. Pilot study not powered for clinical efficacy.

Magnitude and temporal burden of hypotension

The susceptibility of ischemic brain tissue is influenced not only by the severity of the initial perfusion deficit but also by the adequacy and maintenance of collateral circulation until recanalization is achieved. Good collateral status is associated with slower infarct growth and consequently, preservation of brain tissue. However, collateral perfusion may become increasingly pressure sensitive as autoregulatory reserve is exhausted [23]. This vulnerability to intraprocedural hemodynamic fluctuations shifts the attention from absolute BP targets towards the magnitude and cumulative burden of pressure drops before recanalization.

Galecio-Castillo et al. examined whether intraprocedural BP reductions were associated with infarct growth during EVT and how this relationship was influenced by collateral status, assessed using the hypoperfusion intensity ratio (HIR). Hypotensive burden was quantified using hypotensive MAP area (HMA), which incorporates both the magnitude and duration of MAP reduction. Greater HMA was associated with an exponential infarct growth pattern, with a significant interaction between HMA and HIR. Among patients with exponential infarct growth, infarct volume increased by 1 mL for every 100-unit increase in HMA and by 4.2 mL for every 0.1-unit increase in HIR [24]. 

In a retrospective cohort of 390 patients undergoing EVT under general anesthesia or monitored anesthesia care, Petersen et al. examined the relationship between intraprocedural BP changes, infarct progression, and functional outcome. Mean procedural MAP was similar between patients with favorable and unfavorable outcomes (99±15 vs 100±12 mmHg). However, the groups differed significantly in measures of the magnitude of BP reduction (dMAP, dSBP, and mean percentage reductions) and hypotensive burden (hypotensive area for MAP and SBP). dMAP was independently associated with infarct growth and final infarct volume, with an estimated 4.1 mL increase in final infarct volume for every 10 mmHg decrease in MAP during EVT [9]. These observational associations raise the question of whether standardized measures of hypotensive exposure could complement absolute BP targets, particularly under GA, but they do not establish treatment thresholds.

Table 2 summarizes selected studies evaluating the magnitude and temporal burden of intraprocedural BP reduction during EVT. Importantly, dMAP is not a standardized metric across the EVT literature, as studies differ in the reference pressure and procedural BP used to calculate the change. Likewise, area-under-threshold, hypotensive MAP area, and related pressure-time measures share the concept of integrating the magnitude and duration of BP reduction but use study-specific reference pressures and thresholds and should not be considered interchangeable. Figure 1 provides an author-generated schematic illustration of the magnitude, duration, relative reduction, and pressure-time burden of hypotension during EVT. The BP waveform, thresholds, and numerical values are hypothetical and were created solely for conceptual illustration. They are not derived from patient-level data or from any individual study. The concepts illustrated are based on published studies evaluating relative BP reduction and pressure-time burden during EVT [9,24].

**Table 2:** Studies evaluating the magnitude and temporal burden of intraprocedural BP reduction during EVT Study-specific definitions: ¹dMAP = reduction from reference MAP to mean procedural MAP, ²dLMAP/dMAP = reduction from reference MAP to lowest procedural MAP, ³dLMAP = percentage reduction from baseline MAP to lowest procedural MAP, ⁴dMAP = reduction from baseline MAP to each procedural MAP measurement, MAP: Mean arterial pressure, SBP: Systolic blood pressure, aMAP: Area below the respective admission MAP, aSBP: Area below the respective admission SBP, AUT: Area under the threshold, AHA: American Heart Association, BP: Blood pressure, CS: Conscious sedation, dLMAP: Change in lowest MAP, dMAP: Delta MAP, dSBP: Delta SBP, EMS: Emergency medical services, EVT: Endovascular thrombectomy, GA: General anesthesia, HIR: Hypoperfusion intensity ratio, HMA: Hypotensive MAP area, LA: Local anesthesia, MAC: Monitored anesthesia care, MR CLEAN: Multicenter Randomized Clinical Trial of Endovascular Treatment for Acute Ischemic Stroke in the Netherlands, mRS: Modified Rankin Scale

Study	Absolute BP protocol/target	Baseline/reference pressure	Hemodynamic parameter	Study findings and hemodynamic interpretation
Whalin et al., 2017 [25]	SBP target 140-180 mmHg before reperfusion. Anesthetic strategy: MAC	Average of BP measurements before sedation	Lowest value recorded, absolute and % reduction of MAP, SBP from baseline	≥10% MAP reduction, MAP<85 mmHg, and each 10 mmHg MAP decline below 100 mmHg were associated with poor 90-day outcomes.
Treurniet et al., 2018 (post hoc Multicenter Randomized Clinical Trial of Endovascular Treatment for Acute Ischemic Stroke in the Netherlands (MR CLEAN) trial analysis) [26]	GA; no trial-specified BP target	Single brachial measurement at admission	dMAP^1^, dLMAP^2^, absolute and % change	A greater baseline-to-mean MAP reduction was associated with a worse 90-day mRS score; MAP nadir reduction was not.
Petersen et al., 2019 [9]	GA/CS; BP management per treating team/AHA guideline	Earliest MAP charted (EMS/outside hospital/EVT center)	dMAP^2^/dSBP, % BP reduction, hypotensive area (area below the respective admission MAP (aMAP), area below the respective admission SBP (aSBP), mean, lowest and highest BP	Greater dMAP was independently associated with infarct growth, larger final infarct volume, and worse 90-day functional outcome. Greater hypotensive burden (aMAP, aSBP) was also associated with worse outcomes.
Samuels et al., 2021 (MR CLEAN Registry analysis) [27]	CS vs LA	MAP on admission	dLMAP^3^, AUT, procedural BP trend	Greater BP reduction and hypotensive burden were associated with worse outcomes but did not explain the difference in outcomes between CS and LA.
Xu et al., 2021 [11]	GA; BP≤180/105 mmHg	Admission BP	dMAP^4^ >10/15/20/25/30 mmHg, cumulative and longest continuous duration	Each additional minute at dMAP>10 or >15 mmHg increased the odds of poor 90-day outcome by 1.3% and 1.1%, respectively.
Collette et al., 2021 [28]	GA; SBP 140-180 mmHg	MAP 5 min before induction	MAP<70 mmHg or ≥30% below baseline: occurrence, number, and duration of hypotension episodes; AUT, dMAP^2^ analyzed.	Occurrence and frequency of MAP<70 mmHg episodes were associated with worse 90-day mRS. Duration and AUT showed no association.
Galecio-Castillo et al., 2024 [24]	No study-assigned absolute target	Admission MAP	Magnitude of MAP reduction (dMAP^2^), hypotensive burden (HMA), collateral status (HIR), infarct growth	A greater hypotensive MAP area (HMA) was associated with infarct growth pattern, with a significant interaction between HMA and collateral status (HIR).

**Figure 1:**
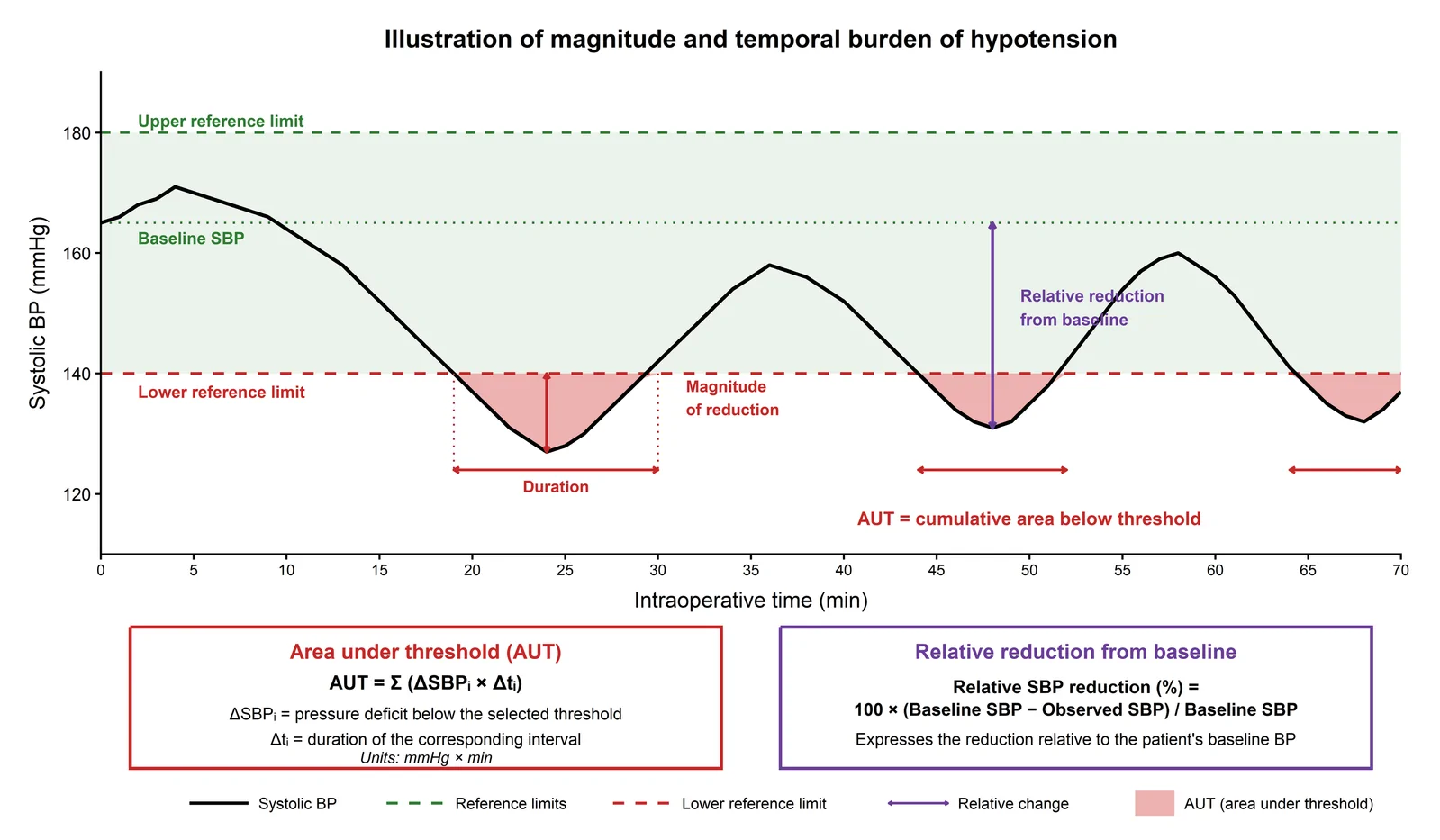
Illustration of the magnitude and temporal burden of intraprocedural hypotension Author-generated schematic demonstrating absolute BP thresholds, relative reduction from baseline, duration of hypotension, and AUT as a measure of pressure-time burden. These metrics are complementary and should not be assumed to represent the same physiological exposure. The BP waveform, thresholds, and numerical values are hypothetical and were created solely for conceptual illustration and do not imply that any single threshold represents an established therapeutic target. No patient-level or study-derived data were used. The concepts illustrated are based on published studies evaluating relative BP reduction and pressure-time burden during EVT [9,24]. The diagram was created by the authors using R statistical software (R Foundation for Statistical Computing, Vienna, AUT). AUT: Area under the threshold, EVT: Endovascular thrombectomy, BP: Blood pressure, SBP: Systolic blood pressure

Blood pressure variability

Blood pressure variability describes fluctuations in arterial pressure over time and can be quantified using overall dispersion, such as standard deviation (SD) or variance; variability relative to the mean, such as coefficient of variation (CV); amplitude of excursions, such as range; or changes between consecutive measurements, such as successive variation (SV) or average real variability (ARV). These metrics capture different statistical properties of the BP trajectory and are not interchangeable [29]. Their values are also influenced by measurement frequency, number of observations, procedure duration, mean BP, monitoring technique, induction, anesthetic depth, vasoactive therapy, ventilation, and fluid administration.

Unlike absolute threshold and pressure-time burden measurements, BPV measures the dynamic dimension of the intraprocedural pressure trajectory. Maïer et al. evaluated the relationship of dynamic BPV and duration below hypotensive thresholds with functional outcome in 172 patients undergoing EVT. Greater SBP, DBP, and MAP variability (CV) was associated with a lower likelihood of a favorable outcome. Among 159 patients with angiographically assessed collateral status, longer exposure to MAP <90 mmHg showed a trend toward worse functional outcome in those with poor collaterals, whereas this relationship was not observed in patients with good collaterals, with a significant interaction between collateral groups [10]. These findings suggest that collateral status may modify the tolerance of the ischemic territory to reductions in systemic pressure.

In contrast, a post hoc analysis of the INDIVIDUATE (Individualized Blood Pressure Management During Endovascular Stroke Treatment) trial found favorable outcomes associated with higher BPV (measured by SBP variance and ARV), whereas time during EVT within ±10 or ±20 mmHg of baseline SBP was not associated with outcome [30]. The discordance across studies may therefore be clinically informative rather than simply contradictory: different BPV metrics, measurement intervals, anesthetic strategies, and patient populations may capture different physiological phenomena. Accordingly, BPV should currently be regarded as a prognostic marker rather than a standardized therapeutic target.

Notably, the literature examining BPV during EVT under anesthesia remains limited compared to postreperfusion BPV studies [31]. These periods represent distinct physiological contexts, particularly before recanalization, when the ischemic penumbra is dependent on collateral perfusion. Additionally, the results of intraprocedural BPV metric studies under GA may not be comparable to those from studies with procedural sedation, as they are influenced by induction, anesthetic depth, positive-pressure ventilation, intravenous fluids, and vasopressor use. Xu et al. demonstrated an independent association between intraprocedural SBP variability (range, SD, CV) and unfavorable functional outcomes in 141 patients exclusively under GA during EVT [32]. Table 3 summarizes selected studies evaluating intraprocedural BPV during EVT. Future prospective studies in well-defined anesthetic cohorts are needed to determine the most clinically informative statistical measures of intraprocedural BPV. Figure 2 provides an author-generated schematic illustration of lower and higher BPV despite similar mean MAP, using commonly reported dispersion-based and successive-change BPV metrics. The figure was created by the authors using R statistical software (R Foundation for Statistical Computing, Vienna, AUT). The BP trajectories, autoregulatory limits, and numerical values are hypothetical and were created solely for conceptual illustration. They are not derived from patient-level data or from any individual study. The concepts illustrated are based on published studies evaluating intraprocedural BPV during EVT [10,30-32].

**Table 3:** Studies evaluating intraprocedural blood pressure variability during EVT ARV: Average real variability, ASTER: Contact Aspiration vs Stent Retriever for Successful Revascularization, BP: Blood pressure, BPV: Blood pressure variability, CS:  Conscious sedation, CV: Coefficient of variation, DBP: Diastolic blood pressure, ETIS: Endovascular Treatment in Ischemic Stroke, EVT: endovascular thrombectomy, GA: General anesthesia, MAP: Mean arterial pressure, mRS: Modified Rankin Scale, NS: Not specified, PP: Pulse pressure, SBP: Systolic blood pressure, SD: Standard deviation, SV: Successive variation

Study	Baseline/reference pressure	Anesthetic strategy	BPV metric	Study findings and hemodynamic interpretation
Jagani et al., 2016 [33]	NS	GA/CS	Maximum SBP, MAP variability	Lower maximum variability in SBP and MAP was associated with favorable outcomes.
Maïer et al., 2018 (ETIS registry) [12]	First BP on arrival to catheter laboratory	GA/CS	Pulse pressure (PP) variability (PP-CV), SBP variability (SBP-CV)	PP variability independently associated with worse 3-month adjusted ordinal mRS.
Maïer et al., 2020 (post hoc ASTER (Contact Aspiration vs Stent Retriever for Successful Revascularization)) [10]	First BP on arrival to catheter laboratory	GA/CS	Dynamic metrics: CV of SBP, DBP, MAP. Steady metrics: hypotension time with SBP <140 mmHg and MAP <90 mmHg	Variability was associated with less favorable outcomes. Hypotension time with MAP <90 mmHg was negatively associated with favorable outcomes in patients with poor collateral status.
Xu et al., 2022 [32]	NS	GA	SD, CV, range and SV of SBP, DBP, MAP	SBP_range_, SBP_CV, _and SBP_SD _were independently associated with unfavorable outcomes. Other BPV indices showed no association.
Chen et al., 2025 (post hoc INDIVIDUATE) [30]	First SBP measured in the emergency room or angiography suite	Sedation	SBP variance, ARV. Time/proportion within ±10 mmHg and ±20 mmHg of baseline	SBP_variance, _SBP_ARV _showed higher odds of favorable outcome. Time within ±10/20 mmHg was not associated with outcome.

**Figure 2:**
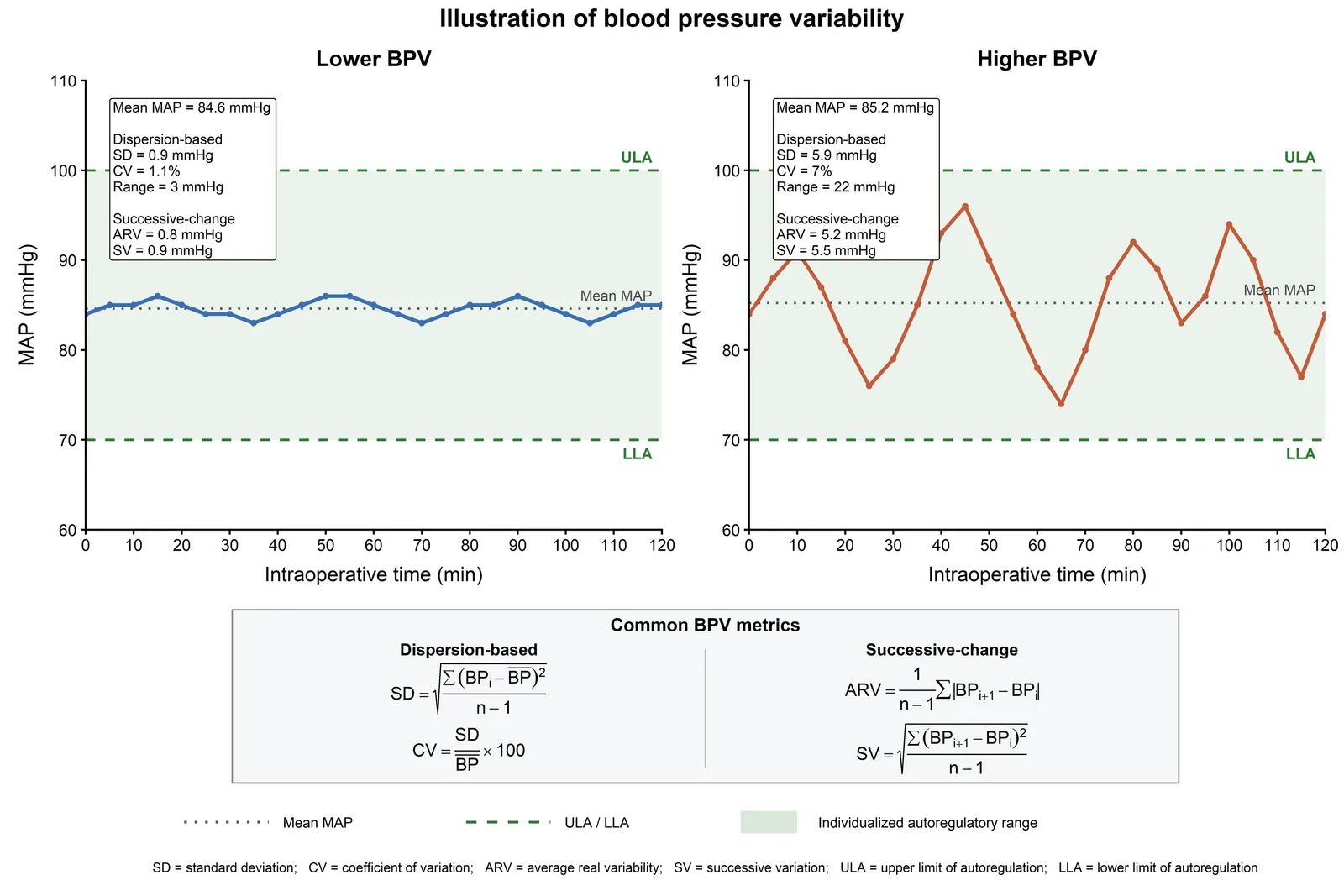
Illustration of BPV Author-generated schematic comparing lower and higher BPV despite similar mean MAP and illustrating commonly reported dispersion-based and successive-change BPV metrics. The BP trajectories, autoregulatory limits, and numerical values are hypothetical and were created solely for conceptual illustration. No patient-level or study-derived data were used. The concepts illustrated are based on published studies evaluating intraprocedural BPV during EVT [10,30-32]. The diagram was created by the authors using R statistical software. BP: Blood pressure, BPV: Blood pressure variability, EVT: Endovascular thrombectomy, MAP: Mean arterial pressure

Individualized cerebral autoregulation assessment

Individualized assessment of cerebral autoregulation refers to assessing the patient-specific arterial pressure range within which cerebral blood flow remains relatively stable despite systemic pressure fluctuations [17]. The limits of autoregulation may vary between individuals and may also be altered by acute cerebral ischemia. During EVT, dCA can be assessed by examining the relationship between fluctuations in systemic pressures and simultaneous changes in cerebral blood flow velocity using transcranial Doppler or in cerebral oxygenation using near-infrared spectroscopy [13]. The absolute thresholds, relative changes, pressure-time burden, and BPV each characterize different dimensions of intraprocedural blood pressure, yet the adequacy of cerebral perfusion cannot be directly established. This provides a physiological rationale for future studies directed towards investigating cerebral autoregulation during EVT for intraprocedural hemodynamic assessment.

The feasibility of cerebral autoregulation assessment has been demonstrated during EVT, although evidence linking autoregulation-derived arterial pressure limits to outcomes remains predominantly post-EVT. Heiberg et al. demonstrated that dCA could be assessed during EVT under general anesthesia using NIRS-derived cerebral oxygenation signals. Autoregulatory characteristics varied between patients and after recanalization, with higher low-frequency gain associated with favorable outcomes and independently predicting functional independence, neurological outcome, and 90-day mortality [34]. The study's outcomes support the potential clinical relevance of assessing patient-specific cerebral autoregulation during EVT. However, individualized arterial pressure limits for intraprocedural blood pressure management were not derived.

Petersen et al. evaluated individualized autoregulatory pressure limits post-EVT by examining the relationship between NIRS-derived tissue oxygenation and changes in systemic MAP. A 10% increase in time spent above the upper limit of autoregulation was associated with a 1.9-fold increase in the odds of a worse functional outcome, whereas fixed BP thresholds were not associated with outcome. Greater time spent below the lower limit of autoregulation was also associated with infarct progression [13]. In a subsequent cohort of 199 patients, Petersen et al. observed a 16.2 mL increase in infarct volume for every 60 minutes spent below the lower limit of autoregulation in non-recanalized patients post-EVT [14].

Although these post-EVT findings cannot be directly extrapolated to the intraprocedural pre-recanalization period, they demonstrate that outcome-relevant arterial-pressure limits may differ between patients and provide a physiological rationale for investigating whether analogous individualized autoregulatory limits are relevant during EVT under anesthesia, particularly while tissue remains non-recanalized. Importantly, an association between an autoregulation-derived metric and outcome does not establish that actively targeting BP to that metric improves neurological outcome. The NIRS- and TCD-based autoregulation assessment also has methodological and physiological limitations, including signal quality and sensitivity to physiological conditions that may influence derived autoregulatory parameters [35]. Accordingly, autoregulatory limits should be regarded as method-dependent estimates that require prospective validation before routine therapeutic use.

Future studies evaluating cerebral hemodynamic metrics under anesthesia should move towards standardized characterization of hemodynamic exposure during EVT. Concurrent evaluation of absolute BP, a clearly defined baseline or reference pressure, the magnitude and duration of hypotensive exposure, and consistently defined BPV metrics and measurement intervals may facilitate comparability across studies and interpretation of apparently discordant findings. Prospective studies incorporating continuous autoregulation assessment during EVT could determine whether pressure-time exposure outside individualized autoregulatory limits provides additional physiological or prognostic information beyond fixed BP thresholds. Figure 3 summarizes the conceptual framework of this review, illustrating how complementary measures of systemic hemodynamic exposure may be interpreted alongside cerebral physiological context during EVT and evaluated in future prospective studies. The diagram was created by the authors using R statistical software as a conceptual synthesis of the reviewed literature. It contains no patient-level or study-derived numerical data and does not represent a validated clinical algorithm.

**Figure 3:**
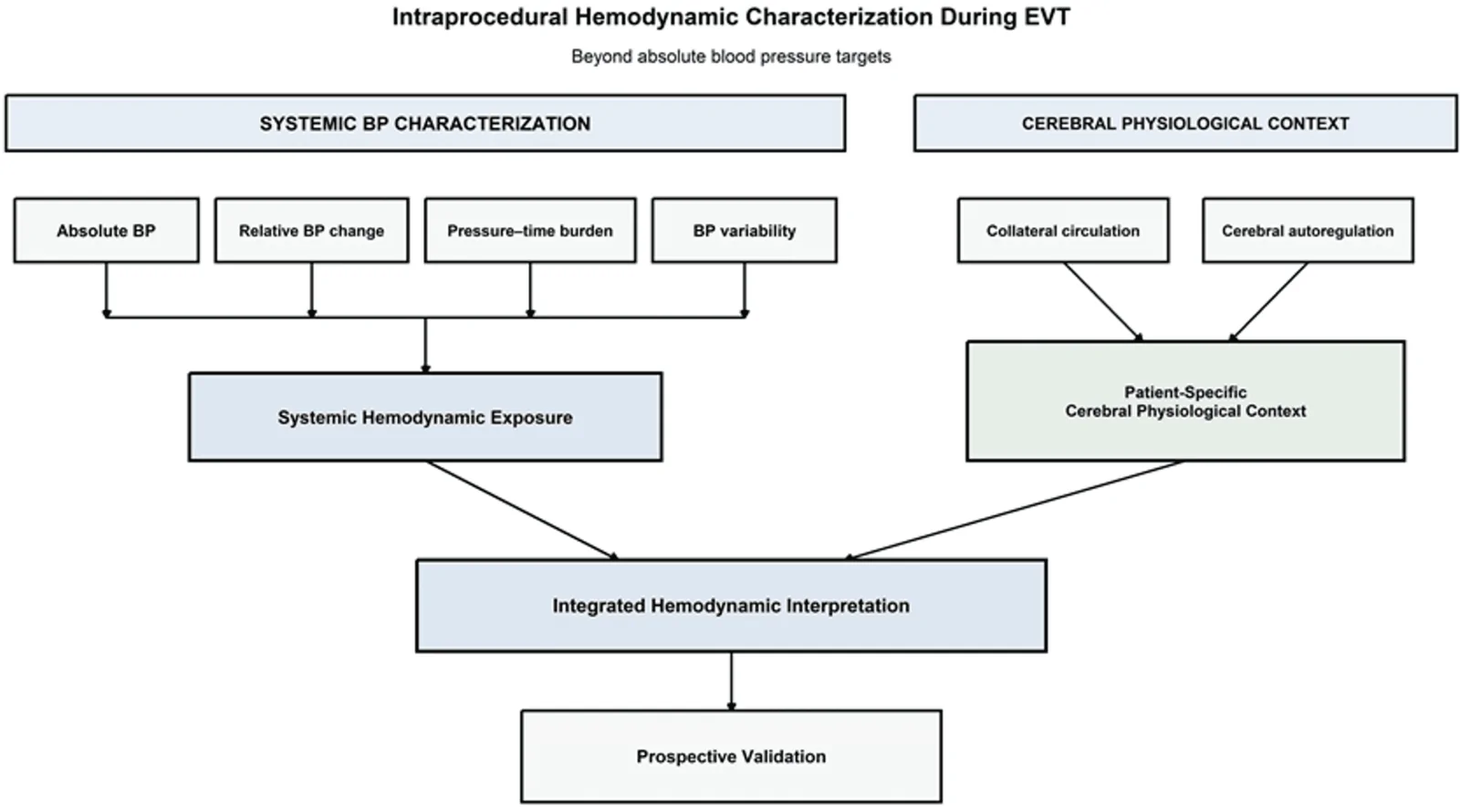
Conceptual framework for intraprocedural hemodynamic characterization during EVT Absolute BP, relative BP change, pressure-time burden, and BPV represent complementary dimensions of systemic hemodynamic exposure but do not directly establish cerebral perfusion adequacy. Collateral circulation and cerebral autoregulation provide distinct cerebral physiological context for interpreting these measures. This author-created conceptual diagram illustrates a framework (created using R Foundation for Statistical Computing) for future prospective evaluation and does not represent a validated clinical algorithm or causal pathway. No patient-level or study-derived numerical data were used. EVT: Endovascular thrombectomy, BP: Blood pressure, BPV: Blood pressure variability

Current evidence supports continued use of established absolute BP targets while recognizing that a single threshold incompletely characterizes intraprocedural hemodynamic exposure. Clinically, substantial or prolonged reductions from a clearly defined reference pressure should be recognized and promptly addressed, and the BP trajectory may provide additional context beyond an isolated nadir. The BPV and pressure-time burden remain prognostic or investigational measures rather than validated therapeutic targets. Individualized autoregulatory thresholds should not yet be used as routine treatment targets outside prospective validation.

Limitations

The findings of this review should be interpreted in the context of its limitations. As a narrative review, study identification and selection were not based on a prespecified search protocol. The primary search was limited to PubMed and predominantly English-language literature. Therefore, relevant studies indexed elsewhere or published in other languages may have been missed. The review focused primarily on intraprocedural blood pressure parameters and did not specifically evaluate the differential effects of anesthetic agents, anesthetic techniques, or vasopressor choice on cerebral hemodynamics and clinical outcomes. Similarly, anterior and posterior circulation strokes were not examined separately, despite potential differences in cerebrovascular physiology and hemodynamic vulnerability. The available evidence also did not consistently permit separation of blood pressure exposure before and after recanalization, although the physiological implications of arterial pressure may differ between these phases. Interpretation is further constrained by the observational and post hoc nature of much of the evidence linking intraprocedural blood pressure parameters to clinical outcomes, which limits causal inference. For example, hypotension may be a consequence of worsening clinical status, longer procedures, anesthetic changes, or treatment of hemodynamic instability rather than an isolated cause of poor outcome. Finally, heterogeneity in the definitions, measurement, and reporting of hemodynamic parameters limits direct comparison across studies.

## Conclusions

Intraprocedural hemodynamics during EVT cannot be fully characterized by a single absolute BP target. Current evidence suggests that relative BP change, hypotensive burden, and BPV provide complementary information about systemic hemodynamic exposure. In clinical practice, these findings support attention to the overall BP trajectory rather than reliance on a single threshold, while recognizing that these measures are not yet validated treatment targets and do not directly determine cerebral perfusion adequacy. Future prospective studies, particularly during GA and before recanalization, should standardize systemic BP characterization and evaluate whether integration with dynamic cerebral autoregulation can identify clinically meaningful, patient-specific hemodynamic strategies.
